# Budesonide/formoterol as effective as prednisolone plus formoterol in acute exacerbations of COPD A double-blind, randomised, non-inferiority, parallel-group, multicentre study

**DOI:** 10.1186/1465-9921-10-11

**Published:** 2009-02-19

**Authors:** Björn Ställberg, Olof Selroos, Claus Vogelmeier, Eva Andersson, Tommy Ekström, Kjell Larsson

**Affiliations:** 1 Department of Public Health and Caring Sciences, Family Medicine and Clinical Epidemiology, Uppsala University, Sweden; 2 Semeco, Ängelholm, Sweden; 3 Universitätsklinikum Giessen and Marburg, Marburg, Germany; 4 AstraZeneca, Södertälje, Sweden; 5 Lung and Allergy Research Unit, Karolinska Institutet, Solna, Sweden

## Abstract

**Background:**

Oral corticosteroids and inhaled bronchodilators with or without antibiotics represent standard treatment of COPD exacerbations of moderate severity. Frequent courses of oral steroids may be a safety issue. We wanted to evaluate in an out-patient setting whether a 2-week course of inhaled budesonide/formoterol would be equally effective for treatment of acute COPD exacerbations as standard therapy in patients judged by the investigator not to require hospitalisation.

**Methods:**

This was a double-blind, randomised, non-inferiority, parallel-group, multicentre study comparing two treatment strategies; two weeks' treatment with inhaled budesonide/formoterol (320/9 μg, qid) was compared with prednisolone (30 mg once daily) plus inhaled formoterol (9 μg bid) in patients with acute exacerbations of COPD attending a primary health care centre. Inclusion criteria were progressive dyspnoea for less than one week, FEV_1 _30–60% of predicted normal after acute treatment with a single dose of oral corticosteroid plus nebulised salbutamol/ipratropium bromide and no requirement for subsequent immediate hospitalisation, i.e the clinical status after the acute treatment allowed for sending the patient home.

A total of 109 patients (mean age 67 years, 33 pack-years, mean FEV_1 _45% of predicted) were randomized to two weeks' double-blind treatment with budesonide/formoterol or prednisolone plus formoterol and subsequent open-label budesonide/formoterol (320/9 μg bid) for another 12 weeks. Change in FEV_1 _was the primary efficacy variable. Non-inferiority was predefined.

**Results:**

Non-inferiority of budesonide/formoterol was proven because the lower limit of FEV_1_-change (97.5% CI) was above 90% of the efficacy of the alternative treatment. Symptoms, quality of life, treatment failures, need for reliever medication (and exacerbations during follow-up) did not differ between the groups. No safety concerns were identified.

**Conclusion:**

High dose budesonide/formoterol was as effective as prednisolone plus formoterol for the ambulatory treatment of acute exacerbations in non-hospitalized COPD patients. An early increase in budesonide/formoterol dose may therefore be tried before oral corticosteroids are used.

**Clinical trial registration:**

NCT00259779

## Background

Chronic obstructive pulmonary disease (COPD) is a major health problem and cause of death and disability. Most patients with COPD suffer from exacerbations, which vary in severity and duration. Frequent exacerbations result in further decline in lung function, quality of life [1,2] and drive mortality [3]. In addition to intensified therapy with bronchodilators, such as β_2_-agonists and anticholinergics, oral corticosteroids represent standard treatment for COPD exacerbations [4]. The value of oral steroids has been widely recognised [5,6] but extending the course beyond two weeks has not provided additional clinical benefit [7]. High doses of nebulized budesonide has also been successfully used for treatment of acute COPD exacerbations in the emergency department [8,9]. Inhaled corticosteroids alone [10] and in combination with long-acting β_2_-agonists [11-14] have been useful in the treatment of patients with moderate to severe COPD, particularly for prevention of severe exacerbations.

In randomized studies in hospitalized patients with acute severe COPD exacerbations, but not requiring ventilation for acute respiratory failure, treatment with budesonide/formoterol in a single inhaler has been compared to inhaled salbutamol on top of a standardized regimen of exacerbation treatment [15] and to i.v. aminophylline and prednisolone plus inhaled salbutamol [16]. The results of these studies indicated that budesonide/formoterol is safe and may be of clinical benefit to COPD patients with acute exacerbations [15,16]. Large, controlled studies were proposed to be performed to evaluate the value of budesonide/formoterol in the treatment of acute COPD exacerbations [17].

Little is known about COPD exacerbations in an out-patient setting. Based on our experience up to 90% of all COPD patients with exacerbations can be treated at primary health care centres and thereafter return home with intensified therapy. It is self-explanatory that these exacerbations are milder than those treated in hospitals or emergency departments. Published clinical studies in patients with COPD exacerbations have been performed in hospitals or in emergency departments. Therefore, we decided to investigate treatment of COPD exacerbations in an out-patient setting and in patients not requiring immediate hospitalisation. To the best of our knowledge this is the first study focused on this type of moderate severe exacerbations. Our hypothesis was that a high dose of the budesonide/formoterol combination could be as effective as prednisolone plus formoterol for this type of acute exacerbations not requiring immediate hospitalisation.

The primary aim of the study was to compare two treatment concepts. The aims were divided as follows: 1) to assess whether 2-weeks' treatment with inhaled budesonide/formoterol (Symbicort^® ^forte Turbuhaler^®^), at a dose of 320/9 μg qid (double the standard dose), is as effective as a 2-week course of oral prednisolone plus inhaled formoterol for the ambulatory treatment of an acute exacerbation, and 2) to assess whether the initial 2-week treatment would influence the rate of exacerbations during a subsequent 12-week open-label treatment period with the fixed combination of budesonide and formoterol at a standard dose of 320/9 μg bid. A non-inferiority design was selected because the aim was not to demonstrate superiority, but rather to investigate whether patients suffering acute exacerbations could be managed as effectively with an inhaled corticosteroid/long-acting β_2_-agonist combination as with the standard oral glucocorticosteroid therapy.

## Methods

### Patients

The target population for this study were current or previous smokers with a smoking history of ≥ 10 pack years, aged ≥ 40 years, with moderate COPD corresponding to GOLD stage IIa or IIb (as defined in GOLD guidelines at the time of initiation of the study) and an established diagnosis of COPD for ≥ 6 months prior to study entry. Study centres were asked to include preferentially patients they had taken care of before. Such patients with an actual acute history of progressive dyspnoea and/or increase in sputum production and/or sputum volume indicative of an acute exacerbation during the week prior to the unscheduled visit to the primary care centre were included if, in the attending physician's opinion, treatment with a course of oral corticosteroids was needed. This excluded patients with mild exacerbations who could be treated with antibiotics and/or an increase in bronchodilator use, and patients requiring oxygen therapy and with a risk for developing respiratory failure and therefore being admitted to hospital for observation and further treatment.

### Study design

This was a double-blind, double-dummy, randomised, non-inferiority, parallel-group, multicentre study performed at 29 primary health care centres and one hospital. All patients received acute treatment with ipratropium bromide and/or salbutamol given by nebulisation or via a pressurised metered dose inhaler attached to a large volume spacer, in combination with a single dose of an oral glucocorticosteroid (prednisolone 30–50 mg or betamethasone 3–8 mg). This treatment was given in accordance with guidelines for ambulatory treatment of acute COPD exacerbations.

After the acute treatment patients were randomised in balanced blocks (sealed envelopes) to the double-blind treatment if their forced expiratory volume in one second (FEV_1_) was 30–60% of predicted normal. FEV_1 _was measured after 15 minutes and up to 4 hours after the acute treatment.

Major exclusion criteria were a diagnosis of asthma, forced expiratory volume in one second/forced vital capacity (FEV_1_/FVC) ratio > 0.7, a previous COPD exacerbation within 30 days prior to the study, oxygen saturation < 92% after the initial acute treatment, requirement for oxygen therapy, a need for immediate hospitalisation as judged by the investigator, treatment with any inhaled corticosteroid in doses > 1000 μg/day at study entry, and use of or need for treatment with a non-selective β-receptor antagonist.

The randomised treatment was budesonide/formoterol (Symbicort forte^® ^Turbuhaler^®^, AstraZeneca, Södertälje, Sweden), 320/9 μg/dose, one inhalation four times daily for 2 weeks, or to prednisolone (Prednisolon Recip, Recip AB, Årsta, Sweden), 30 mg once daily, plus inhaled formoterol (Oxis^® ^Turbuhaler^®^, AstraZeneca, Södertälje, Sweden), 9 μg twice daily for 2 weeks. Patients receiving regular anticholinergics at study entry were allowed to continue taking that medication during the entire study period. Antibiotics could be prescribed if judged necessary by the investigator.

Following the 2-week double-blind treatment period all patients were treated with open-label budesonide/formoterol, 320/9 μg one inhalation twice daily for additional 12 weeks. Exacerbations, defined as worsening of COPD requiring a course of oral steroids or hospitalisation, were recorded. The first patient entered the study in September 2005 and the last subject completed it in July 2007.

### Assessments

The patients visited their health care centre for assessment at the end of weeks one and two, and at the end of the open follow-up period. The primary efficacy variable was the change in FEV_1 _from baseline to treatment for one and two weeks measured at the health care centre. Other efficacy variables were treatment failures, i.e. the number of patients requiring additional medication due to disease deterioration during the first 2 weeks, FEV_1 _measured twice daily at home with a Piko-1^® ^electronic peak flow meter (Medica Pharma, Uppsala, Sweden), the number of patients with an exacerbation and the time to first exacerbation during the follow-up period. Patients also recorded daily symptoms (difficulty to breathe, cough, chest tightness and night-time awakenings) on a scale from 0 to 4 and use of reliever medication (ipratropium bromide, Atrovent^®^, inhalation powder, 40 μg per dose, Boehringer Ingelheim). A self administered Clinical COPD Questionnaire (CCQ) [18] was completed at the start of the study, after one week and at the end of the double-blind period. Total individual scores on a scale from 0 to 6 were calculated, as were scores for the subgroups physical function, mental health and symptoms. A difference of 0.4 was considered clinically important [19].

At all visits serum C-reactive protein (CRP) concentrations were measured using a latex immunoassay (Aeroset^® ^CRP Vario, Abbott Scandinavia AB, Solna, Sweden).

Safety was monitored by reporting of adverse events, serious adverse events and discontinuations due to adverse events.

The study was performed according to Good Clinical Practice and the Declaration of Helsinki. All local ethics committees approved the study protocol. All patients gave their written informed consent for participation.

### Determination of sample size

FEV_1_, being a robust endpoint in an ambulatory setting, was used for power calculation. The sample size was calculated to show non-inferiority of budesonide/formoterol. The null hypothesis was that mean values for the new and the standard treatments were not equivalent and that the lower limit of the one-sided 97.5% confidence interval (CI) of budesonide/formoterol was not below 90% of the effect shown with the standard treatment. With a sample size in each group of 43 completed patients, the power was 80% with the common standard deviation of 0.170 of the log-transformed FEV_1_.

### Statistical analysis

The intention-to-treat principle was used, i.e. all patients who received at least a single dose of study medication were included in the analyses.

The primary analysis was a non-inferiority test as described above in the sample size calculation. The predefined non-inferiority limit of 90% was selected to be more demanding than the 80% to 125% limits often used when comparing pharmacological responses. The analysis was performed by log transforming the FEV_1 _% predicted values using an analysis of covariance (ANCOVA) model with the treatment and country as factors and log-transformed baseline FEV_1 _% of predicted as a covariate. The least-squared means resulting from this model were used to calculate the one-sided 97.5% CI for the log-transformed difference between the two treatments.

The secondary variables, CCQ and FEV_1 _measured at clinic visits, were analysed by calculating the 95% CI for the adjusted mean differences. The secondary diary card variables, morning FEV_1_, evening FEV_1_, intake of study drug, use of reliever medication and COPD symptom scores were analysed using an analysis of variance (ANOVA) with treatment and country as factors. A non-inferiority test was not used since that would have required predefined non-inferiority limits for each variable.

The number of patients with treatment failures during the 2-week double-blind period were compared between treatments. The analysis of exacerbations in the 12-week open-label period was based on the number of patients with exacerbations using a log-rank test.

Changes in serum CRP concentrations and safety data were analysed using descriptive statistics.

## Results

### Study population

A total of 113 patients were randomised and 109 were included in the analyses of efficacy and safety. The patient characteristics and demographics at baseline are shown in Table 1. The budesonide/formoterol group included more female patients than the prednisolone plus formoterol group. No other important differences between the study groups were observed, including both previous maintenance medications and acute treatment before study entry.

**Table 1:** Patient baseline demographics and characteristics

	**Budesonide/formoterol** **N = 55**	**Prednisolone + formoterol** **N = 54**
**Age, years**	67.2 (9.7)	66.7 (9.3)
**Females, %**	55%	43%
**Time since COPD diagnosis, years**	8.0 (5.7)	5.9 (4.3)
**Current smokers, %**	33%	28%
**Pack-years (range)**	33 (10–120)	33 (10–83)
**Body mass index**	25.2 (4.8)	26.0 (5.3)
FEV*, L	1.16 (0.34)	1.23 (0.37)
FEV_1_*, % predicted normal	45.1 (8.9)	45.0 (9.5)
FVC*, L	2.42 (0.73)	2.48 (0.70)
FEV_1_/FVC* ratio	0.49 (0.12)	0.51 (0.11)
**GOLD classification (no. of patients)**		
**I**	1	1
**IIa**	28	33
**IIb**	26	18
**III**	0	2
**SpO_2_**	95.2 (1.7)	94.9 (1.6)
**Maintenance medication at study entry^†^:**		
**ICS + LABA + AC**	24	20
**ICS + LABA**	10	9
**ICS + AC**	1	5
**AC ± SABA/LABA**	14	7
**No medication**	6	13
**Mean dose of ICS,^‡ ^μg/day**	659 (267)	553 (241)

Data are presented as means and standard deviations. * Lung function measurements were performed 0.25–4 hours (mean 0.9 hours) after acute therapy; ^† ^ICS = inhaled corticosteroid; LABA = long-acting inhaled β_2_-agonist; AC = anticholinergics, SABA = short-acting inhaled β_2_-agonist; ^‡ ^Mean ICS intake in patients with recorded ICS use.

Compliance analyses, based on the patients' diary card recordings during the 2-week double-blind period, revealed that the patients in the budesonide/formoterol group used an average daily dose of 1151 μg of budesonide of 1280 μg prescribed and 32.0 μg of formoterol of 36 μg prescribed. The patients in the prednisolone plus formoterol group used 29.0 mg of prednisolone of 30 mg prescribed and 17.3 μg of formoterol of 18 μg prescribed.

### FEV_1_

**Figure 1:**
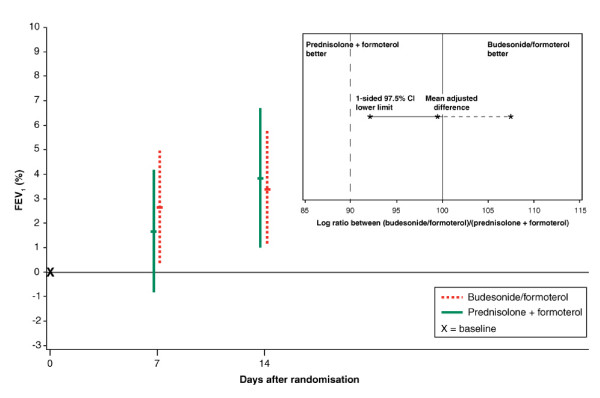
**Change in FEV_1 _as % predicted, measured at the clinic, from baseline to one and two weeks for all patients (main graph) and log ratio between treatments (inset graph)**. Data are presented as means and ± 95% CIs. * The treatment with budesonide/formoterol was non-inferior to the standard treatment with prednisolone plus formoterol because the predefined limit of at least 90% effect with budesonide/formoterol was superseded by the value (92.0%) of the lower limit of the 97.5% CI. The mean effect of budesonide/formoterol was 99.4% of standard treatment and the upper 97.5% CI limit was 107.4%.

The changes from baseline in clinic FEV_1_, as percentages of predicted values, showed non-inferiority for combination inhalation therapy (budesonide/formoterol) compared with oral/inhalation treatment (prednisolone plus formoterol) (Figure 1). Non-inferiority was also observed when analysed separately after treatment for 1 week and 2 weeks, respectively (Figure 1). FEV_1 _and FEV_1 _% predicted at baseline and after treatment for one and two weeks are shown in Table 2 as well as the percent change in FEV_1 _after treatment for one and two weeks.

**Table 2:** FEV_1 _at clinic visits and Clinical COPD Questionnaire (CCQ) scores during the 2-week double-blind treatment period

**Variable**	**Time point**	**Budesonide/formoterol**	**Prednisolone + formoterol**	N*
**FEV_1_, L**	Baseline	1.17 (0.35)	1.20 (0.36)	49/51
	1 week	1.25 (0.45)	1.23 (0.34)	49/51
	2 weeks	1.27 (0.43)	1.29 (0.40)	49/51

**FEV_1_, change from baseline, %**	1 week	6.84 (23)	2.50 (24)	49/51
	2 weeks	8.55 (24)	7.50 (29)	49/51

**FEV_1_, % predicted**	Baseline	44.8 (9.2)	44.4 (9.4)	49/51
	1 week	47.4 (12.1)	46.0 (11.3)	49/51
	2 weeks	48.3 (12.0)	47.9 (12.4)	49/51
**CCQ, score 0–6**	Baseline	3.30 (0.95)	3.35 (1.02)	45/51
	1 week	2.67 (1.25)	2.58 (1.11)	45/51
	2 weeks	2.52 (1.19)	2.32 (1.11)	45/51

Data are presented as means and standard deviations. * Refers to patients in the budesonide/formoterol and prednisolone + formoterol groups, respectively with recordings at all visits.

The change in FEV1 % predicted from baseline was statistically significant already at 1 week in the budesonide/formoterol group (p = 0.03), but not in the prednisolone plus formoterol group (p = 0.17). After 2 weeks this change was significant in both groups; budesonide/formoterol (p = 0.01) and prednisolone plus formoterol (p = 0.005).

**Figure 2:**
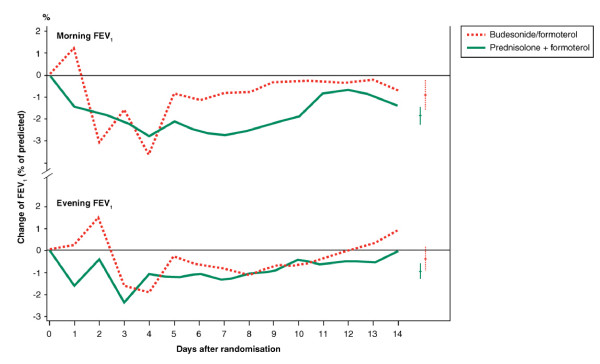
**Change in FEV_1 _as % of predicted from daily measurements at home during the double-blind period**. Bars to the right are presented as means and ± 95% CIs.

Daily morning and evening FEV_1 _values during the 2-week double-blind period, measured at home with the Piko-1 meter, are shown in Figure 2. No improvements from baseline (first measurement at home in the morning and evening respectively) were seen in either group. There were no statistically significant differences in FEV_1 _between the two groups and the shapes of the curves over the 2-week period look very similar.

### Treatment failures

There were two treatment failures in the budesonide/formoterol group (one patient after 1 day requiring hospitalisation for 5 days, and another after 5 days not requiring hospitalisation). In both cases social factors not related to the disease or randomized treatment appear to have been involved. No treatment failures were reported in the prednisolone plus formoterol group.

### Use of reliever medication

On average, during the 2-week double-blind period, the patients in the budesonide/formoterol and prednisolone plus formoterol groups used 1.8 and 2.1 inhalations per day of reliever medication, respectively. The difference was not statistically significant. Separating the number of reliever inhalations into day-time and night-time inhalations yielded similar results.

### COPD symptoms

**Figure 3:**
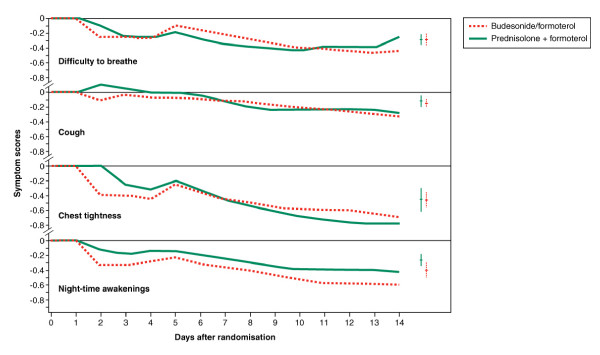
**Change from baseline in four COPD symptom scores during double-blind treatment: difficulty in breathing, cough, chest tightness and night-time awakenings**. Bars to the right are means and ± 95% CIs. Lower values represent improvements.

There were no statistically significant differences in symptom scores between the two groups (Figure 3).

### Clinical COPD Questionnaire

The changes in total CCQ scores from baseline to week 1 and 2 visits were -0.72 and -0.9 in the budesonide/formoterol group and -0.77 and -1.09 in the prednisolone plus formoterol group, respectively (Table 2). There were no statistically significant differences between the groups; neither in total CCQ scores, nor in subgroup scores for function, mental health and symptoms.

### Exacerbations

**Figure 4:**
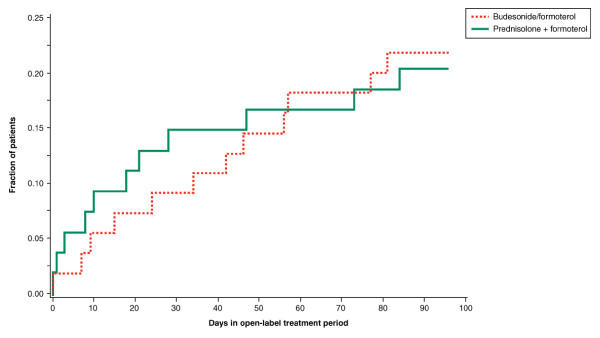
**Time to first exacerbation during the open-label treatment period**. Day 0 corresponds to the end of the double-blind treatment period. The groups shown represent the treatment arms for the double-blind treatment period.

Neither the number of exacerbations nor the time to first exacerbation differed significantly between the groups during the 12-week open-label period, when all patients received budesonide/formoterol. There were 11 patients with 14 exacerbations (one hospitalisation) among 55 patients in the budesonide/formoterol group and 10 patients with 14 exacerbations (three hospitalisations) among 54 patients in the prednisolone plus formoterol group. The time to the first exacerbation is shown in Figure 4.

### C-reactive protein concentrations

**Figure 5:**
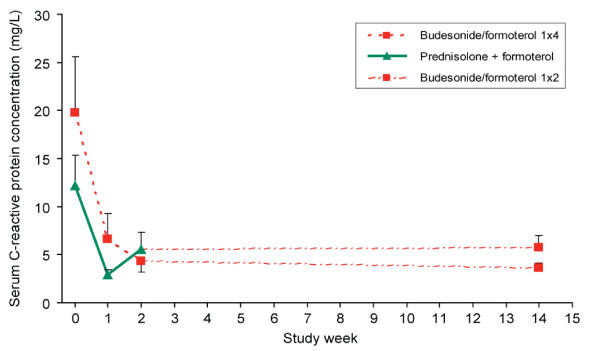
**Serum C-reactive protein levels at baseline and after one, two and 14 weeks' treatment**. Data are presented as means and standard errors of the mean. Between week two and week 14 all patients were treated with budesonide/formoterol, 320/9 μg, one dose twice daily.

Mean serum CRP levels at baseline were higher in the budesonide/formoterol group than in the prednisolone plus formoterol group (19.8 mg/L, SEM 5.8 versus 12.2 mg/L, SEM 3.2). After both the 2-week double-blind period and 12-week open-label period the mean serum CRP levels were similar in both groups (Figure 5).

### Safety

No safety concerns were raised in the study. In the budesonide/formoterol group 15 patients had 18 adverse events and 14 patients had 15 adverse events in the prednisolone plus formoterol group. These adverse events represented a variety of preferred terms and were similar in the two groups.

## Discussion

In the present study two treatment concepts for ambulatory patients with COPD exacerbations were compared. Out-patients making unscheduled visits to the health care centre because of an acute moderate COPD exacerbation not requiring immediate hospitalisation, but judged by the investigator to require a course of oral corticosteroids, were selected for the study. This study demonstrated that budesonide/formoterol, taken four times daily and initiated at the time of onset of the exacerbation was as effective as standard treatment with oral prednisolone plus formoterol.

The baseline lung function data, smoking histories, the fact that the patients were well known to the investigators and the exclusion of patients with a diagnosis of asthma strongly support the COPD diagnosis in the included patients. An exacerbation of COPD has been defined as a sustained worsening from a stable state and beyond normal day-to-day variation, which is acute in onset and may necessitate a change in regular medication [20]. In long-term clinical trials a variety of different definitions for COPD exacerbations have been used [21]. However, in studies dealing with acute exacerbations as an inclusion criterion the definition is generally based on the patient's clinical history during the past few days. The patients in our study fulfilled clinical COPD exacerbation criteria as they all reported increasing symptoms prior to the unscheduled first study visit to the primary care centre. The occurrence of an exacerbation is supported by the elevated serum CRP levels at the time of randomisation and a poor quality of life as previously described during an acute exacerbation [22]. In fact, the mean CCQ score at onset of the exacerbations was slightly worse in our study (mean CCQ score 3.3) than in the study by Bourbeau et al who reported a mean CCQ score of 3.0 [21].

It could be argued that the lack of a placebo arm is a limitation of this study and that a similar effect of the two treatments may reflect a lack of a clinical effect of either treatment. However, as it has been clearly and indisputably shown in placebo-controlled trials that glucocorticosteroids are beneficial in patients with COPD exacerbations [4-6,23,24], it was considered unethical to include a placebo group in the present study. As this study included only patients who had a deterioration of their clinical status during the last week prior to entry, and as there was a subsequent improvement in all evaluated parameters and a decrease in initially elevated CRP levels, we believe that there was a true clinical effect in both groups.

It could also be discussed whether all treatment effects were due to the initial single dose of oral steroid plus the nebulisation of bronchodilators. However, for safety reasons we found it hard not to give this treatment as the aim was to send all patients home for further randomized treatment and not to consider hospitalisation. Secondly, this acute treatment was given to all patients and it appears unlikely that a carry-over effect would have lasted for the duration of the study.

The comparator treatment in our study was oral prednisolone plus inhaled formoterol. We considered the duration of the prednisolone course (2 weeks) to be adequate because prolongation of prednisolone therapy to eight weeks has previously been shown to add no clinical benefit [7].

There was an improvement in FEV_1 _of 8–9% and in FEV_1 _predicted normal of approximately 3% in both groups. The improvement was already statistically significant at 1 week with budesonide/formoterol and after 2 weeks for both treatments. The small improvement in FEV_1 _could be regarded as clinically irrelevant, but it should be noted that the observed improvements in FEV_1 _were on top of baseline values obtained after acute treatment with nebulised bronchodilators and systemic corticosteroids at study entry. In addition to similar effects on lung function in both groups, several secondary and clinically more relevant efficacy variables, such as symptoms, quality of life and need for reliever medication, were found to be equally improved in the budesonide/formoterol group and the prednisolone plus formoterol group. The occurrence of two treatment failures in the budesonide/formoterol group does not alter this conclusion as these were clearly unrelated to the randomized treatment.

The doses of inhaled formoterol differed during the double-blind period; patients in the budesonide/formoterol group used a mean of 32 μg per day, whereas the patients in the prednisolone plus formoterol group used on average17.3 μg per day. It is possible that the higher formoterol dose may have resulted in a more pronounced effect on lung function since a dose-response relationship for FEV_1 _in stable COPD patients has been demonstrated with formoterol [25]. However, it is not known whether a dose-response applies also for treatment of exacerbations. As the formoterol doses of both treatment groups were rather high it seems reasonable to assume that the difference in doses probably had no or only a marginal influence on the results.

The results of our study can be compared to the results of the only so far reported study with budesonide/formoterol in COPD patients and acute exacerbations, although reported only as an abstract [16]. This study was performed in hospitalized patients with baseline mean FEV_1 _values around 0.85 L. The patients in our study were out-patients with mean FEV_1 _values above 1.15 L after acute treatment with a single dose of oral prednisolone plus nebulized bronchodilators, and from the beginning considered able to return home after the acute treatment. Therefore, our patients may have represented a milder type of COPD exacerbations. However, as the patients' baseline per cent of predicted FEV_1 _values are unknown in the study by Cazzola et al [16] it is hard to more precisely compare the study populations. The efficacy, reported at 72 hours, of budesonide/formoterol 160/4.5 μg × 4 every 6 hours measured as improvements in FEV_1 _was statistically significant in the study by Cazzola et al [16] and not different from the improvements in the comparator group receiving aminophylline 240 mg and prednisolone 20 mg i.v. every 12 hours plus inhaled salbutamol 400 μg every 6 hours. However, a comparison of the effect level is also difficult as we report results after treatment for one and two weeks and the observation time in the study by Cazzola et al was 72 hours [16].

The main purpose of using an inhaled medication as one of the treatment options was the idea of testing a medication that in the future could be part of a self-management plan for out-patients with not too severe exacerbations. The difference in the way the corticosteroid component was administered may have played an important role for the results. Although the daily dose of budesonide was not higher than 1280 μg (delivered dose via Turbuhaler corresponding to 1600 μg of a metered dose) this dose in patients with stable COPD has been found very effective [26]. From a safety point of view, however, this dose causes significantly less effects on e.g. the pituitary-adrenal axis than the comparator dose of 30 mg prednisolone [27].

A clinically valuable observation from our study was that the dry powder inhaler, Turbuhaler, functioned well in patients with acute COPD exacerbations. This observation supports the results of an earlier study, performed in an emergency room setting, where patients with COPD responded equally well to formoterol administered via Turbuhaler and formoterol given using a pressurised metered dose inhaler attached to a spacer [28].

Based on the results of earlier studies [7,22,29] we considered a 3-month follow-up period to be sufficient to evaluate the incidence of further exacerbations. Time to first exacerbation and the number of exacerbations during the follow-up period, when all patients were treated with the approved standard dose of budesonide/formoterol, were almost identical in the two groups, showing that the initial budesonide/formoterol treatment did not influence the long-term outcome compared with initial treatment using prednisolone plus formoterol.

CRP is recognised as a marker of systemic inflammation [30] and patients with worsening of COPD have higher serum CRP levels than healthy control subjects [31]. In this study, CRP at baseline was higher in the budesonide/formoterol group than in the prednisolone plus formoterol group, probably indicating more severe systemic inflammation in this group. The decrease in serum CRP observed in the budesonide/formoterol group cannot be explained by the high formoterol doses because β_2_-agonists do not influence serum CRP levels [32]. The demonstration that budesonide/formoterol and prednisolone plus formoterol decrease serum CRP levels to the same extent is in agreement with an earlier study which showed a marked reduction in serum CRP levels in stable COPD patients treated with inhaled corticosteroids [32]. The equivalence of the CRP decline in the two treatment groups is suggestive of equal efficacy of the two treatments.

The risk of systemic side effects when using oral prednisolone – even short courses – has been well recognised [33] and the total steroid burden may be heavy in patients with frequent exacerbations. Short-term increases in the doses of inhaled budesonide have been found safe and well tolerated [27,34]. Thus, from a safety standpoint, treatment of acute COPD exacerbations using inhaled medications would constitute a clear advantage over therapy with oral corticosteroids.

## Conclusion

Treatment of acute exacerbations of non-hospitalized COPD patients with an elevated dose of the fixed combination of budesonide/formoterol is as effective as a standard treatment with an oral corticosteroid plus formoterol. Being a safe alternative by avoiding courses of oral steroids, treatment with budesonide/formoterol in patients with these moderate severe COPD exacerbations may therefore be a clinically important alternative for physicians working in primary health care centres. Carefully designed controlled trials should be performed to assess whether increasing the dose of the fixed combination, early in the course of symptom deterioration, is beneficial when used as part of a self-management approach.

The study was funded by AstraZeneca

## Competing interests

Björn Ställberg has during the last five years been paid for lectures and for consulting from AstraZeneca, GlaxoSmithKline, Boehringer Ingelheim and Pfizer.

Olof Selroos has during the last five years been a consultant to AstraZeneca and received fees from pharmaceutical companies including AstraZeneca for medical writing (including this manuscript) and speaking at meetings.

Claus Vogelmeier has given presentations at industry symposia sponsored by Altana, AstraZeneca, Aventis, Bayer, Boehringer, GlaxoSmithKline, Merck Darmstadt, Talecris. He has also received fees for consulting from Altana, AstraZeneca, Bayer, Boehringer, Glaxo Smith Kline, Talecris

K Larsson has during the last five years on one or more occasions served in an advisory board, as speaker, and/or participated in educations arranged by AstraZeneca, Boehringer Ingelheim, GlaxoSmithKline, MSD and Pfizer. K Larsson has also received unrestricted research grants from Boehringer Ingelheim, GlaxoSmithKline and AstraZeneca during the last five years.

Eva Andersson and Tommy Ekström are employees of AstraZeneca.

## Authors' contributions

The study was designed and the protocol developed by BS, OS, TE and KL. EA was responsible for coordinating the study. BS and KL worked as principal investigators. The results were interpreted by BS, OS, CV, TE and KL. The manuscript was drafted by OS. All authors gave substantial critical input in revising the manuscript.
